# Patient perceptions of cognitive screening in adult audiology services: A qualitative exploration

**DOI:** 10.3389/fneur.2023.1143128

**Published:** 2023-04-03

**Authors:** Emma E. Broome, Puntrika Tannirandorn, Jean Straus, Phoebe Beale, Eithne Heffernan, Tom Dening, Helen Henshaw

**Affiliations:** ^1^National Institute for Health and Care Research (NIHR) Biomedical Research Centre, Nottingham, United Kingdom; ^2^School of Medicine, University of Nottingham, Nottingham, United Kingdom; ^3^Nursing, School of Health Sciences, University of Nottingham, Nottingham, United Kingdom; ^4^Mental Health and Clinical Neurosciences, School of Medicine, University of Nottingham, Nottingham, United Kingdom

**Keywords:** cognitive screening, adult aural rehabilitation, hearing loss, cognitive impairment, qualitative research, mild cognitive impairment

## Abstract

**Introduction:**

Both hearing loss and dementia are extremely pervasive, especially amongst older adults. As hearing loss and dementia have common symptoms, misdiagnosis can be common, and failure to address hearing loss for people with dementia could accelerate cognitive decline. The timely detection of cognitive impairment is clinically important, however the use of cognitive assessments in adult audiology services is a hotly debated topic. Although the early detection of cognitive impairment may improve patient care and quality of life, patients attending audiology services for hearing assessment might not expect to be asked questions about their cognition. The aim of this study was to qualitatively explore patient and public perspectives and preferences on the use of cognitive screening within adult audiology services.

**Methods:**

Quantitative and qualitative data were gathered from an online survey and a workshop. Descriptive statistics were applied to quantitative data and an inductive thematic analysis was performed on free-text responses.

**Results:**

In total, 90 respondents completed the online survey. Overall, cognitive screening in audiology was reported to be acceptable to participants (92%). A reflexive thematic analysis of the qualitative data reported four themes: i) knowledge of cognitive impairment and screening, ii) implementation of cognitive screening, iii) impact of screening on patient and iv) contributions to future care and research. A workshop was held with five participants to discuss and reflect on the findings in more detail.

**Discussion:**

Participants found cognitive screening to be acceptable within adult audiology services providing audiologists had suitable training, and sufficient explanation and justification were provided. However, implications such as additional time and staff resource and supplementary training for audiologists would be required to address participants concerns.

## 1. Introduction

Hearing loss is a major public health issue. The World Health Organization (WHO) estimates that globally, 466 million adults have disabling hearing loss with numbers projected to rise to 700 million by 2050 (1). In the United Kingdom (UK), one in five adults are affected, which makes it the second most common disability in the UK (2). The prevalence and severity of hearing loss increases with age. More than 40% of people aged over 50 years live with hearing loss, increasing to more than 70% of those aged 70 years or older (3). Restrictions in communication arising from hearing loss can affect an individual's interpersonal relationships, educational and career opportunities as well as their ability to interact with services, including healthcare. The combination of all these factors can affect psychological health and wellbeing through social and emotional withdrawal (4–6). Additionally, the estimated risk of dementia for those with untreated hearing loss compared to those without is twice as likely for those with mild hearing loss, three times greater for those with moderate hearing loss and five times greater for those with severe hearing loss (7). Ultimately, hearing and communication difficulties can have a significant impact on an individual's quality of life.

Cognition refers to the mental processes involved in acquiring knowledge and comprehension through thought, experience, and the senses (8). Cognitive abilities describing the change in function over our lifespan are categorized into crystallized and fluid intelligence. Crystallized intelligence refers to skills that are well-practiced and familiar such as vocabulary and general knowledge (9). These remain stable or gradually improve over time, peaking in the late 60s to early 70s (10). On the other hand, fluid cognition signifies a person's innate ability to process, learn and manipulate new information (10, 11). Examples include executive function, processing speed, memory, and psychomotor ability. Fluid cognitive abilities typically peak in the third decade of life and exhibit a continuous decline into the later years of life (10). Hearing, or listening in noise, relies on peripheral hearing, central auditory processing and cognition (e.g., attention and working memory) (12, 13). Cognition plays a role in listening, with greater working memory and attention skills associated with better speech in noise understanding for people with hearing loss (14, 15).

Dementia is an umbrella term used to describe the progressive and gradual decline in cognitive function with severe effects on social and physical activities. It has been estimated to affect almost one million adults in the UK, rising to 1.6 million people by 2050 (16). The symptoms of dementia can vary depending on the cause, but its main clinical manifestations can be categorized into cognitive and psychological changes. Cognitive changes can include difficulties in communication, visual and spatial abilities, reasoning, problem solving, coordination, memory loss and confusion or disorientation (17). On the other hand, psychological changes include personality changes, depression, anxiety, inappropriate behavior, paranoia, agitation, and hallucinations (18). Mild cognitive impairment (MCI) is the earliest stage of dementia and approximately 80% of MCI patients develop dementia within 6 years of diagnosis (19).

Current hypotheses suggest that there are three main possible mechanisms through which hearing loss is associated with cognitive decline (20). First, in individuals with a hearing loss, greater cognitive resources may be necessary to process auditory signals, thus increasing cognitive load and depleting cognitive reserve (21). Second, some studies suggest structural changes in the brain structure of individuals with hearing impairment (22). Finally, both hearing loss and cognitive decline are independently associated with social isolation (23). Another possibility is that both hearing loss and cognitive impairment are caused by a common mechanism, or that the association is multifactorial (24). Global populations are aging at an unprecedented rate and numbers are expected to accelerate in the coming decades (25). Society will be required to adapt and restructure across all sectors to accommodate for the shift in age demographics (26). There are over 11 million people aged 65 and over in the UK and this will have increased to 13 million people or 22% of the population in the next 10 years (27). With the increase in number and proportion of aging individuals, the number of people affected by both dementia and/or hearing loss is also expected to increase. Hearing loss has been identified as the largest modifiable mid-life risk factor for dementia (28). As hearing loss is highly prevalent and can be managed through aural rehabilitation, early detection of hearing loss represents an opportunity to address potentially causal mechanisms of cognitive decline (24).

Hearing loss and cognitive impairment can present similarly. This can cause increased difficulty in distinguishing the true cause of these symptoms over time. Examples of overlapping symptoms include short-term memory problems, difficulty in understanding and following conversations and social withdrawal (23, 29–31). Individuals with cognitive impairment/dementia commonly have trouble processing speech in the presence of competing background noise and may also struggle to express their hearing difficulties or communication challenges. Family or carers may misinterpret these difficulties as related to dementia rather than a potentially correctable hearing problem (32, 33). The combination of these factors can present challenges and then cause misdiagnosis and delay in presentation to healthcare professionals. This may further delay provision of the correct treatment and have a greater impact patient's quality of life. Ongoing studies are investigating whether addressing hearing loss, by providing amplification or alternative intervention can prevent, slow, or reverse cognitive decline in individuals with hearing loss (34–36). In the United States, the Aging and Cognitive Health Evaluation in Elders (ACHIEVE) randomized controlled trial, is the first to evaluate the efficacy of a best-practice hearing intervention in delaying cognitive decline in older adults with untreated hearing loss (37). A recent meta-analysis reported that the use of hearing devices in individuals with hearing loss was significantly associated with a reduction in cognitive decline and an improvement in cognitive testing scores (38). However, evidence remains inconclusive (21, 39, 40) due to lack of longitudinal research (41).

Cognitive screening tests are short tests which can be used to assess how well the brain is functioning. They are designed to test our cognition (or thinking abilities), such as memory, language, judgement, and the ability to learn new things. Such tests comprise part of the assessment of possible dementia but are not in themselves sufficient to make the diagnosis due to their lack of specificity. There are many other reasons for low scores on cognitive testing, for example they are usually verbally administered, as such people with hearing loss tend to perform worse than individuals with normal hearing (42). However, some cognitive screening tests have been adapted for people with hearing loss for example by removing verbal items or presenting items visually (43, 44). Nonetheless, cognitive testing is usually regarded as an essential tool, to either to raise the possibility of a cognitive disorder or to quantify its degree. It has been proposed that cognitive screening tests could be used in audiology clinics to aid early detection of cognitive impairment or dementia for onward referral and support. Such tests could guide audiological care through interventions such as hearing aid programming and follow up. There are many factors which need to be considered before cognitive screening could be implemented in audiology services. For example, consideration should be given to the purpose of the screening, how it would be conducted, any necessary training and the procedure for onwards referral with health services, this list is by no means exhaustive. In addition to any practical and clinical considerations, it is important to understand whether cognitive screening is acceptable to patients attending adult audiology services.

This study aims to explore patient perceptions of cognitive screening delivered within UK adult audiology services by analyzing free-text responses from an online survey and a workshop.

## 2. Materials and methods

### 2.1. Study design

This study reports a qualitative analysis of free-text answers from an online survey of 90 participants in the UK and a workshop.

### 2.2. Ethical approval

Ethical approval was obtained from the University of Nottingham Faculty of Medicine and Health Science Research Ethics Committee (FMHS 438-0122). All participants provided informed electronic consent.

### 2.3. Participants and recruitment

Participants were included if they were (i) aged 18 years or older, (ii) had a diagnosis of dementia or mild cognitive impairment and/or a hearing condition (e.g., hearing loss), or (iii) were receiving care as a person living with dementia or mild cognitive impairment and/or a hearing condition or (iv) were a carer/communication partner providing support to someone who is living with dementia/mild cognitive impairment and/or a hearing condition.

It is important to consider the perspective of stakeholders such as carers or family members of people living with hearing loss and/or dementia who may support the individual to attend clinical appointments and complete tests. Thus, this research includes the perspective of key stakeholders including both carers and communication partners of people living with dementia and/or hearing loss.

Participants were recruited using purposive sampling; this included contacting participants from the National Institute for Health and Care Research (NIHR) Nottingham Biomedical Research Center (BRC) research participant database *via* email and through using social media channels (e.g., Twitter and a blog post). The first authors (EB, PT) monitored the sample during data collection (45) and recruitment ceased when data saturation was obtained (46). Data saturation occurred when the first authors (EB, PT) identified no new patterns pertinent to the research question within the online survey responses (47).

### 2.4. Data collection

#### 2.4.1. Online survey

Participants completed a 15-min questionnaire through an online platform (JISC Online Surveys, https://www.onlinesurveys.ac.uk). First, participants were provided with an electronic Participant Information Sheet with a description of the study's aim and the researchers contact details in case participants wished to ask any questions or had concerns. Next, participants completed an electronic consent form and provided basic demographic data. Participants were asked to complete three questions regarding their opinions on the use of cognitive screening in adult audiology services. Participation was voluntary and participants could withdraw at any time by exiting the online survey without giving a reason.

The questions were developed by the research team (EB, PT, HH) in consultation with a Patient Research Partner (JS) to consider how they might be interpreted by the participants. The questions are listed in Box 1.

Box 1 **Online survey questions.**

1. If you went to get your hearing tested, would you be happy for someone to ask you questions about your memory?
2. How would this make you feel?
3. Do you have any other thoughts you would like to share with us?

The first question was a close-text response; possible response options were “Yes” and “No”. The second and third questions allowed participants to reflect on their personal perceptions of cognitive screening in adult audiology services. The analysis focused on the free-text responses to the open-ended questions. Data were collected between 21st September and 5th October 2022.

#### 2.4.2. Workshop

Participants who completed the online survey were invited to register their interest to attend an online workshop to explore the findings from the online survey in greater depth. The workshop provided an opportunity to explore the topic of cognitive screening in audiology services, using two different data collection methods (i.e., a survey and a workshop). The collection of data about the research question using different methods is a form of qualitative triangulation, which is an established strategy for enhancing the rigor of qualitative research (48). The workshop took place on 14th November 2022, for two hours, and was facilitated by the first two authors (EB and PT) and consisted of two steps. First, workshop participants were asked to reflect on the online survey questions listed in Box 1. Second, participants were asked to review and respond to the findings of the qualitative analysis of the online survey by reflecting on the themes and sub-themes from the reflexive thematic analysis. The process of data triangulation allowed participants to reflect and elaborate on their survey responses and provide in-depth feedback on the survey themes. The workshop was video recorded online using Microsoft Teams and transcribed verbatim to capture the findings. The analysis process is described below.

### 2.5. Patient and public involvement

A Patient Research Partner (JS), an individual with lived experience of hearing loss, was involved in the design and conduct of this research. JS contributed to writing the blog post, reviewing the online survey questions, and the content of the workshop. She also provided comments on the final manuscript prior to submission.

### 2.6. Data analysis

Demographic information and information collected from close-ended questions were analyzed using descriptive statistics. Anonymised identification codes were assigned to the survey participants (e.g., SP1) and the workshop participants (e.g., WP1). Written informed consent was obtained from study participants for the publication of anonymous direct quotes.

A reflexive thematic analysis of the free-text responses to the online survey was conducted following Braun and Clarke's (49–52) six-step process on the free-text responses from the online survey. This method was chosen as it offers a flexible yet robust and well-established system to gain a detailed account of qualitative data. The process followed is detailed in Table 1.

**Table 1:** Phases of thematic analysis.

**Step**	**Phases**	**Description of the process**
1	Familiarizing yourself with your data	Transcribing data (if necessary), reading and re-reading the data, noting down initial ideas
2	Generating initial codes	Coding interesting features of the data in a systematic fashion across the entire data set, collating data relevant to each code
3	Searching for themes	Collating codes into potential themes, gathering all data relevant to each potential theme
4	Reviewing themes	Checking if the themes work in relation to the coded extracts (Level 1) and the entire data set (Level 2), generating a thematic “map” of the analysis
5	Defining and naming themes	Ongoing analysis to refine the specifics of each theme, and the overall story the analysis tells, generating clear definitions and names for each theme
6	Producing the report	The final opportunity for analysis. Selection of vivid, compelling extract examples, final analysis of selected extracts, relating back of the analysis to the research question and literature, producing a scholarly report of the analysis

The first author (EB) a researcher with expertise in dementia and hearing loss research, who has formal training in qualitative methods and first author (PT), a medical student, independently familiarized themselves with the free-text responses of the full data set and developed a list of initial codes. All initial codes were collated using Microsoft Excel. Any responses containing multiple meanings was assigned as many codes as appropriate. After completion of their independent lists, both researchers (EB and PT) discussed and reviewed each coding decision together. Discrepancies were resolved through discussion until a consensus was reached. Subsequently, the two authors met to generate and refine the overarching themes and subthemes. Themes and sub-themes were then checked against the raw data to ensure they represented the participants' responses. Data summaries were presented to the research team members (JS, EH, HH, TD) as part of a peer debriefing process to discuss insights obtained from the survey and to refine the qualitative analysis. Subsequently, all authors (EB, PT, JS, EH, PB, HH, TD) provided feedback on the narrative.

#### 2.6.1. Workshop analysis

The workshop provided an opportunity to explore the online survey questions in greater depth with a sub-set of participants. Specifically, participants at the workshop engaged in member reflection, which entailed reviewing and providing feedback on the survey themes and reflecting and elaborating on their survey responses (53). Data from the workshop were analyzed using a primarily deductive thematic approach. According to Braun and Clarke (52, 54), thematic analysis is conducted at a point on the continuum between primarily inductive analyses, which prioritize data-driven meaning, and primarily deductive analyses, which prioritize analyst-based or theory-based meaning (55, 56). Deductive approaches can be used to explore the evidence for, explicate, and amend existing themes from previous research (57, 58). Even primarily deductive analyses often use inductive elements, such as inductive coding, generating inductive subthemes within deductive themes, or generating both inductive and deductive themes (55, 57–59). In the present study, the primarily deductive analysis was used to explore the themes derived from the online survey data in greater depth and to identify any additional themes stemming from the workshop data. The analysis entailed applying codes from the online survey analysis to the workshop data, as well as generating new codes for any workshop data that did not conform to the pre-existing codes. This analysis was conducted by two members of the research team (PB and EH) using Microsoft Word. EH was a researcher with expertise in hearing loss research and qualitative methods and PB was a nursing student.

## 3. Results

### 3.1. Demographic profile

The demographic profile of survey takers is shown in Table 2. Fifty-four participants (60%) were female with an overall mean age of 66.6 years ± 14.1. Of the 90 survey participants, 82 (92%) individuals self-reported as living with a hearing condition (e.g., hearing loss and/or tinnitus) and four (4%) as living with a cognitive condition (e.g., mild cognitive impairment). Of the sample, 10 participants identified that they were a carer of someone living with a cognitive condition and two reported being a communication partner of someone living with hearing loss.

**Table 2:** Participant characteristics.

	**Online survey**	**Workshop**
Number of participants	90	5
**Age**		
25–34	6	-
35–49	6	-
50–64	32	1
≥ 65	46	4
Mean (SD)	66.67 (14.14)	75.20 (11.34)
**Sex**		
Male	36	3
Female	54	2
Male:Female	1:1.5	1.5:1
**Ethnicity**		
White (British, Irish, Other White Background)	87	5
Asian or Asian British	2	-
Mixed	1	-
**Occupation**		
Full-time employed	22	-
Part-time employed	11	-
Part-time carer	1	-
Retired	51	4
Student	1	-
Other	4	1
**Level of education**		
Secondary school up to 16 years	15	1
Higher or secondary or further education	18	2
College or University	38	-
Post-graduate degree	19	2

### 3.2. Disposition toward cognitive screening in audiology clinics

Overall, the majority of survey takers (83 participants, 92.2%) indicated that they were willing to be screened for cognitive impairment in an adult audiology clinic.

### 3.3. Qualitative analysis

Four themes were identified describing patient perceptions of cognitive screening in adult audiology services: (1) knowledge of cognitive impairment and screening; (2) implementation of cognitive screening; (3) impact of screening on patient; and (4) contribution to future care and research. Each of these themes comprised several subthemes (Table 3). Generally, the themes were derived from the online survey data and were supported by the workshop data. One additional subtheme was generated through the analysis of the workshop data (Subtheme 2.4).

**Table 3:** Themes/sub-themes.

**Theme**	**Subtheme**
1. Knowledge of cognitive impairment and screening	1.1. Awareness and perceived risk of cognitive impairment 1.2. Early detection and intervention
2. Implementation of cognitive screening	2.1. Understanding and justification of screening 2.2. Delivery of screening 2.3. Patient concerns about cognitive screening 2.4. Relationship with the audiologist
3. Impact of screening on patient	3.1. Emotional associations with screening 3.2. Holistic care 3.3. Interest in cognitive screening
4. Contribution to future care and research	4.1. Future care and research 4.2. Professional awareness and training

#### 3.3.1. Theme 1: Knowledge of cognitive impairment and screening

The first theme refers to participants' existing knowledge of the relationship between hearing loss and cognitive impairment, the implications of using cognitive screening for the early detection of cognitive impairment and the consequences of untreated hearing loss.

##### 3.3.1.1. Sub-theme 1.1: Awareness and perceived risk of cognitive impairment

Most participants reported that they were aware of the link between untreated hearing loss and the impact this may have on cognitive impairment. Participants reported how this knowledge related to how acceptable they felt cognitive screening in adult audiology services to be:

*I'm happy to do this as I've read magazine articles about the impact hearing loss can have on long term cognitive function*. SP53

*I've been aware of this idea that there's a…potential link between your levels of hearing and cognitive function…My consultant said to me… “If you delay with hearing aids…the part of your brain that's involved in hearing…it's not being used, so it kind of dies away”. So, for me, I think I'd be quite happy to be asked questions about memory in screening*. WP4

Sources of knowledge regarding this topic included authoritative sources, such as the Royal National Institute for Deaf People (RNID) and healthcare professionals.

However, the suggestion that hearing loss could be linked to dementia was viewed negatively by several participants:

*I wouldn't particularly like it. Two reasons. 1. I don't like the implied assumption that hearing impairment leads to memory loss. 2. Also, it's something I have never considered. I don't like the thought of such a possibility being planted in my mind*. SP26

*Scared about the future, as I understand there are links to hearing loss and early-onset dementia*. SP3

This particularly related to the fear and stigma surrounding developing cognitive impairment or dementia.

*One the main disadvantages is you're going to worry about it…I'd want to…reassure people that it's not necessarily a bad thing, but…you might well trigger something in someone by not knowing…their…own personal experiences…of dementia*. WP4

Nonetheless, some participants viewed cognitive screening as a reasonable precaution for people whom they perceived to have potential risk-factors, such as those with a family history of dementia, past medical history and/or being of a certain age.

*I've always experienced hearing loss…but I've never come across the fact that there was a relationship between that and cognition and that would probably have been very helpful a few years ago if I knew that there were something of that nature happening, particularly when you get into your 80s, you become more aware that that there is possibly something that could be…related to it*. WP5

*Having got to [a certain age], I'm beginning to feel that things like memory are important and that we need to…keep an eye on ourselves and…friends of similar ages to find out if problems are occurring*. WP3

However, some participants raised the issue that cognitive screening would perhaps not be appropriate for people of a younger age:

*At what age would…you suggest that this started? Would it end up being anybody who has a hearing loss is tested for cognitive impairment? I have a son…and he's very proud of his hearing aid…but I think he would be very, very put off by the thought of having cognitive testing [at] his age*. WP3

##### 3.3.1.2. Subtheme 1.2: Early detection and intervention

One of the reported benefits of cognitive screening was the ability to detect cognitive impairment earlier on in the care pathway, thus enabling access to potential treatment:

…*any cognitive impairment could be picked up and mitigated to some extent, as soon as possible. So, I would be glad of the questions*. SP34

*The sooner dementia is diagnosed the better the chances of treatment*. SP38

*I never, ever…thought that hearing loss would be associated with a cognitive impairment…People should be made more aware of that rather than wait until it's too late and by the time you actually get a…diagnosis, you may well be in the stages where you're not aware enough to actually do anything about it*. WP1

Receiving an earlier diagnosis of cognitive impairment and subsequent pathway to treatment was viewed positively by those who reported personal experiences of caring for someone living with dementia:

*Since I was carer for my mother who had Alzheimer's I would be only too pleased to be assessed because the earlier the treatment the better if any is needed*. SP64

*Having looked after my late husband with Parkinson's/ Lewy body dementia, my feelings would only be positive that it may contribute to earlier diagnoses*. SP6

One workshop participant described their personal experience of dementia and hearing loss, and how the symptoms of both conditions often masked each other:

*My father-in-law…[had] dementia and…hearing problems…It was very, very difficult at times to find out whether it was his hearing aids that were playing up…or whether it was actually…dementia…I would be personally quite happy for anybody to try and link one with the other or to isolate one from the other…If they can isolate that you haven't got the hearing problem and it is…dementia related, I think you're actually removing one of the…obstacles for forward treatment*. WP1

Participants tended to regard hearing loss as readily mitigated but, in contrast, did not offer any suggestions as to what might help the management of cognitive impairment.

*It's much more important to deal with the hearing loss than it is to worry about cognition…Whatever you do about tracking cognition, hearing loss is what you can do something about and…it's much more prevalent*. WP2

#### 3.3.2. Theme 2: Implementation of cognitive screening

Many responses related to how cognitive screening could/should potentially be implemented within an adult audiology service. These mainly focussed on interactions between patients and audiologists during a hearing appointment, particularly when discussing cognitive testing, and how cognitive screening would fit into the patient care pathway for example how the results might be used. Patients also raised several concerns about the practical implementations of cognitive screening.

##### 3.3.2.1. Subtheme 2.1: Understanding and justification of screening

Most responses highlighted the importance of providing an adequate explanation and justification of why cognitive screening was being conducted, to patients, prior to administering any test:

*If it were done without explanation, I'd be confused, and probably feel insulted. However, if there were a reason given which made sense in the context of the appointment then that would be fine*. SP38

*Without a succinct explanation it would be a matter of trust rather than seeing a benefit to myself or others*. SP86

*If you give a decent explanation, which is the fact that…there is a…link between hearing loss and dementia, then people will [be] very happy to answer those straightforward questions*. WP2

The explanation of cognitive screening was viewed as particularly important; participants described how they did not associate a hearing assessment with anything relating to cognition. Therefore, it would be necessary for the audiologist to take the time to carefully explain and justify why cognitive screening tests needed to be conducted. Without this aspect some participants reported that they would feel apprehensive about the screening. As one participant put it “*the attitude of the questioner is key”* (SP27). Two workshop participants recommended that audiologists frame cognitive screening in a way that emphasizes the positive aspects and that minimizes alarm.

*If it's pitched [as] screening, it's no different from having your blood pressure checked…If you can pitch it in such a way that people understand it as part of a general health screening, rather than something that's specific to them [so] that they don't feel picked upon…It's not something that…they're exhibiting as such…It's part of a general screening that's preventative that [is available to] everyone who comes within the orbit of the audiology department*. WP4

*I went recently for general health screening test and…the phrase that was used…was maintaining active independence, so it was positioned for me as a positive thing… I think the way that you present these tests as being something that…you can be in control of…is much more positive than the idea that it's going to…identify something that's wrong with you, so the presentation of it…is really key*. WP4

One participant stated that they would like the opportunity to ask the audiologist questions about the screening. Although participants emphasized the need to understand *why* the screening was being conducted, some still stated that they would feel concerned about the outcome of the results if they indicated that the patient had any impairment.

##### 3.3.2.2. Subtheme 2.2: Delivery of screening

The delivery of cognitive screening manifested in two different ways. First, participants emphasized the mode of delivery of the screening tests, for example, if they were to be delivered orally:

*It would be interesting to find out if the tests are via audio [and] if they allow for the hearing loss, and taking time to hear and process the request. Vs. for example visual or written tests*. SP46

Reference was made to the impact hearing loss may have on a verbal cognition test, for example responding incorrectly if unable to hear or mishearing the questions asked. As one participant noted “*if the patient cannot understand the test, how can you make a satisfactory diagnosis?*” (SP3). One workshop participant emphasized the importance of having a short yet informative cognitive assessment.

*It desperately depends what questions you've been asked…The full standard test for…mild cognitive impairment is…quite a long-winded process: 20 minutes or so. I presume that that is not the sort of thing you are proposing…The whole of this discussion does depend…on a…relatively short and simple and screening process… Are there meaningful tests [that are] relatively brief?* WP2

*I don't understand how you could get a short cognitive test that would be…meaningful…This process needs to start by…defining…the possible tests…Having participated in cognitive testing…it's not a short process…Its impact is significant…on the patient…Let's hear about the cognitive test, which could be at all sensibly added into [a hearing assessment]*. WP2

Similarly, another workshop participant recommended that cognitive testing should be incorporated into hearing assessments to ensure the process is informative and streamlined.

*If there's some way of putting [cognitive testing] in with an audiology test…Once something has been devised that will tie the two together, at least from the audiologist side of things, they can roll out that. [Then we can say] “It's not a hearing problem that's causing the lack of understanding”…which points it toward the other way…When you have the GP test of [cognition], it doesn't rule out the hearing side…An audiologist…[is] in a position to say that… “Your hearing is fine. There is another problem.”* WP1

Finally, it was highlighted how participants were keen to know what would happen to result of the screening tests and the potential referral pathway onwards if the tests indicated a certain level of impairment. It was suggested that patients should have surety that they would receive further practical support and input from either General Practitioners (GPs) or memory clinics, if required.

*We can get different [clinicians] each time…I have found, because I've had many operations in my time, is that the way that the computer system works within the health service…as long as the data goes to a GP…then it can be very helpful in the way things are processed from there*. WP5

*I would quite like to be given some…information to take away with me at that point…I have always…managed my own hearing loss…so I'm quite used to doing that…[I would prefer] feedback…in person on the day, or at least some information about where…these results might lead…because the idea that it might go via the GP is…probably good if you have a proactive GP, if you can get an appointment, if you can access them. But otherwise, I think it's good if you…have an awareness yourself*. WP4

##### 3.3.2.3. Subtheme 2.3: Patient concerns about cognitive screening

Concerns about cognitive screening centered on two primary areas: (i) the qualifications of the person administering the test and (ii) the accuracy of the test. Patients suggested that audiologists may require additional training to deliver this type of testing, as they felt that cognition would not be their primary area of expertise. In addition, cognition, or cognitive impairments, were highlighted by respondents as being a condition associated with stigma. Therefore, audiologists would have to be equipped with the necessary skills to be able to discuss and communicate with patients about this topic in a sensitive way.

*Only if the audiologist had been suitably trained in dealing with a very sensitive topic*. SP38

In some circumstances, participants felt that additional input would be required from healthcare professionals outside of the audiology department, for example from a GP. One participant noted:

*If I was worried about my memory, I would ask the doctor, or blank it. I would not want anyone asking me about memory whilst testing my ears. If you had lost some hearing that is bad enough without me thinking that the tester is thinking I have lost my memory as well*. SP36

In addition, several participants described how they would want any concerns to be followed up by the “*professionals qualified to help*” (SP1).

It was reported that sensory impairment, such as hearing loss, could influence the result of the cognitive screening test, as certain conditions may mask each other. Moreover, participants living with hearing loss described how listening fatigue impacts their ability to process and answer questions accurately. As discussed previously, an inability to hear an oral cognitive test will likely impact the result. Some participants emphasized how this could result in misleading assumptions about patients' cognition:

*My hearing [loss] results in a lot of information in conversations being incomplete and or inaccurate as I rebuild and guess at missing words. So poor memory can be seen as the issue where my memory is ok but the original information, I heard is inaccurate. Someone not recognizing this could make incorrect assumptions resulting in a poor and misleading diagnosis*. SP28

One participant described how having a carer attend alongside could provide additional information, rather than solely relying on patient reports or measures.

##### 3.3.2.4. Subtheme 2.4: Relationship with the audiologist

Several workshop participants felt it was important for the audiologist to establish a good relationship with the patient, including developing a sense of trust and understanding, before carrying out cognitive testing.

*My initial reaction would be that…[cognitive testing] could be useful, but it depended very much on what the audiologist or whoever I was talking to was like and how much I felt they understood the situation. It's a big leap of faith in a way, isn't it?* WP3

Two workshop participants noted that cognitive testing could be detrimental to audiologist-patient relationship and deter patients from attending audiology appointments, especially if the testing is not handled in a sensitive manner.

*I know nobody who has got dementia who didn't have worries about it a long before they were in any sense properly diagnosed and I think there is a significant danger…to be dealt with that…by asking the question…you'll turn them off audiology*. WP2

*I also have a…friend who's deaf, who is absolutely terrified of the audiologist and…that sort of testing would probably push her over the edge of never going back to an audiologist…, which would make the original problem much worse...You do have to be very careful about the questions you ask*. WP3

It is crucial to ensure that cognitive testing does not deter patients who may already be reluctant to have their hearing assessed and managed due to the considerable stigma associated with hearing loss and hearing aids.

*What you've got here really is a…double whammy in that there's so much negativity around…hearing loss in general that it's…seen still as a kind of a weakness. People don't think twice about wearing glasses now, but they would think twice about wearing hearing aids…You almost [need to get] over that…negativity about hearing loss before you can even deal with…the cognitive…loss as well, so I can see why people will just kind of run away screaming from…the idea of either of them*. WP4

A barrier to establishing sufficient trust is the lack of continuity such that patients rarely encounter the same audiologist across different appointments.

*You just cannot get that continuity where the person you've perhaps spoken to in the first place did understand the problem that you were describing, then you've got to start completely again with somebody who may not pick it up on the same wavelength*. WP1

#### 3.3.3. Theme 3: Impact of screening on patient

The majority of participants reported an emotional reaction to the thought of cognitive screening. Most of the emotions had negative associations but, despite this, some participants described how they were interested in the results of the screening and could understand how it could contribute to a holistic view of care.

##### 3.3.3.1. Subtheme 3.1: Emotional associations with screening

Many participants reported a strong emotional reaction to the thought of cognitive screening being conducted within the context of an adult audiology hearing appointment. Participants described how they would feel irritated as they felt questions about cognition did not relate to the purpose of their visit to audiology:

*I'd probably feel slightly irritated if I was asked questions which did not relate to the purpose of my visit*. SP7

Issues of cognitive impairment provoked feelings of concern and worry in many participants. This manifested as “*embarrassment*” about failing the test and thus being perceived as lacking cognitive capability. In addition, emotions such as “*worry*”, “*anxiety*” and “*apprehension”* were mentioned with respect to the screening test potentially uncovering a cognitive impairment:

*Scared about the future, as I understand there are links to hearing loss and early-onset dementia*. SP33

Nonetheless, a small number of participants reported that cognitive testing would have no impact on them at all.

*The diagnosis…of cognitive impairment…brings enlightenment, if I can put it that way…It's better to know than not know, whatever it may be*. WP2

##### 3.3.3.2. Subtheme 3.2: Holistic care

Some participants considered that screening cognition could contribute to providing holistic care, by considering the assessment of more than one condition. This presented in two ways, first that screening could be used as an “*indicator for current or future health issues*” (SP7). Second, that it could provide “*a more rounded image of health and cognition*” (SP58), rather than focussing on health conditions independently.

*Your symptoms that…you're worrying about…may not be to do with cognitive impairment. They may just be do something like stress or…hearing so that [cognitive testing] can be positioned as something that is reassuring as much as it's diagnostic*. WP4

*[Through cognitive testing] you can look at the way that it's impacting your life and get…tips at the initial stages…for how you can deal with…early cognitive impairment and prevent things like depression becoming an issue, so…it can be helpful to know this as a way of being prepared and also to…avoid mental health issues*. WP2

It was suggested that an understanding of a patient's cognition could help the audiologist when speaking to patients about their hearing loss:

*Establishing a patient's ability regarding memory loss might help the audiologist when talking to a patient about their hearing loss*. SP38

Some participants described how the results of the screening could be monitored at each hearing appointment to detect changes over time. Additionally, one respondent suggested that screening cognition during hearing appointments may be a way of detecting people who are reluctant to go their GP to raise any potential issues about cognitive impairment.

##### 3.3.3.3. Subtheme 3.3: Interest in cognitive screening

Despite the emotional reactions reported by participants, a common view was that they would be interested in having their cognition screened:

*I would be interested to find out more about my memory and how it compares to others of my age if any testing is being done*. SP70

This was mainly described in the context of having results of the test conveyed to patients at the time of screening. Some participants stated that they would be “*curious”* about the results and “*would be happy to receive any results or find out if problems are showing*” (SP57). One workshop participant suggested that it would raise awareness of any cognitive problems, and thus patients would be in a better position to manage it:

*I've never even really heard of the word [cognition] before this…If you're not aware of any problem, then you can't deal with it so…if somebody said to me “You've got a slight problem now and this is the way you ought to deal with it”…that would be extremely helpful*. WP5

The same participant described how patient awareness of cognitive problems, through screening in audiology, would be useful on both a personal and public level:

*I think it's got to be a very positive thing because…it's about other people having the perception of what is wrong with you. That becomes helpful even to the person who's suffering*. WP5

#### 3.3.4. Theme 4: Contribution to future care and research

A common theme of participants was that cognitive screening could contribute to their future care, both within audiology and the health and social care system more generally. Reference was also made to the potential to impact future research.

##### 3.3.4.1. Sub-theme 4.1: Future care and research

The acceptability of cognitive screening was related to participants' perceptions that the results could benefit either themselves in the short term, by identifying additional health concerns and/or access to treatment, or others by contributing to future research. One survey participant suggested that the results of the cognitive screening could be recorded and used in “*future examinations*” (SP12). Potential avenues for future research included a better understanding of the link between hearing loss and cognition:

*If the answer was to further the understanding of health conditions/my condition and might lead to the development of new/improved treatments or it was for tailoring existing treatment for my condition, I would feel very pleased that I had contributed*. SP60

##### 3.3.4.2. Sub-theme 4.2: Professional awareness and training

Several workshop participants advised that an important direction for future care and research is to raise awareness of hearing loss and hearing aids. For example, one participant stated that there should be greater knowledge amongst the public of hearing loss and dementia as both individual and co-existing conditions.

*One of the other really important things about…the contribution to future care and research is just the general raising of awareness…through either articles in the press and the media…Then throughout the population you have…increased awareness of…these issues as individuals, but also the combined impact of the two issues together*. WP4

Three workshop participants reported that care home staff need greater awareness of and training in hearing loss, including an understanding of how it can affect many people living with dementia.

*Those of us here who have hearing loss…know it's important but within the care system, it's a relatively smaller thing…Training, training, training of the care home system is what is needed…far more than worrying about diagnosing people with cognitive impairment at hearing tests…Probably a very significant proportion of people in care homes with dementia are…suffering from age-related hearing loss…It comes back to this training, training, training within the care home system*. WP2

It is particularly important for care home staff to have the knowledge and skills to carry out hearing aid maintenance. However, training alone may be insufficient. They also need the resources and facilities to support hearing aid use and maintenance, such as readily available supplies of batteries.

*One of the problems we had with my mother-in-law when she was in a care home was that nobody actually understood how hearing aids worked and regularly they were left in the drawer and there were no batteries in them…That can be a very, very, very big problem and I don't know how you solve that, because even if you train people, they forget the next day*. WP3

*[My] father-in-law eventually went into a care home…He was wearing hearing aids…but we'd go in there and we would think he's just looking blank…[The] hearing aids… had batteries that just hadn't been changed…Within the care system…little things like hearing aid batteries and the tubes…if they're not checked regularly, then these [residents] that they've got both the cognitive problem and the hearing problem are sitting in a room just looking at other people, day in and day out…There should be a system within the care system for them to be checked and tested…and at least had batteries available for them*. WP1

## 4. Discussion

This study qualitatively explored patient perceptions of conducting cognitive screening in adult audiology services. It found that overall, cognitive screening was acceptable to most participants. However, there were some caveats concerning the implementation of cognitive screening in clinical practice. These centered around the qualifications and experience of the audiologist in delivering cognitive tests, conveying the results to participants and the potential implications for future care for example, onwards referral to primary care. The relationship and trust between the audiologist and patient could also play an important role in ensuring that patients feel comfortable with cognitive screening.

The acceptability of cognitive screening, appeared to be linked to participant awareness of the link between cognitive impairment and hearing loss (7). In addition, participants' age, or their perception of aging, was related to their views on the appropriateness of cognitive screening. For example, older respondents highlighted the known relationship between cognition and aging, thus were more likely to report positive views of cognitive screening compared to younger respondents. The Lancet commission on Dementia (28) highlighted that untreated hearing loss is the largest mid-life modifiable risk factor for dementia. Further research is needed to understand the potential benefits of detecting hearing loss in mid-life and fitting of hearing aids to ascertain whether intervention can improve or delay cognitive decline.

In addition, recent international practice recommendations for the management of hearing and vision impairment in people living with dementia advocates for improving the awareness and knowledge of the implications of comorbid sensory and cognitive impairment with both the public and healthcare professionals (60). As hearing loss and dementia are both progressive conditions, cognitive screening in adult audiology services could offer an opportunity to monitor an individual's level of cognitive function over time, as suggested by participants in this study. Some researchers have suggested the development of auditory cognitive stress tests' to detect early stages of neurodegeneration (61). Identifying untreated hearing loss in individuals with cognitive impairment could have benefits including improved communication and quality of life (62). It may be that people living with cognitive impairment require additional support or adaptions in order to use their hearing aids. It remains an open question as to whether earlier intervention for hearing loss could help to prevent or delay cognitive impairment. There are encouraging signs in the literature (63), but there is a lack of prospective research to demonstrate such benefits. In any case, in order to be able to undertake such studies requires early identification of hearing problems. The findings in this study suggest that patients feel that audiologists may require further specialist training to explain, administer, interpret and discuss cognitive screening tests with patients. This study supports evidence which emphasizes the importance of trust between audiologists and their patients (64), and would be pertinent when discussing potentially a sensitive topic such as cognitive impairment. Previous research suggests that audiologists typically focus on hearing aids, spending less time addressing psychosocial concerns in patients (65). In particular, patient-centered communication and shared decision making have both been identified as areas for improvement for audiologists (66). Barriers to addressing psychosocial concerns, such as loneliness, have been suggested to include a lack of time, training and continuity (67).

Consistent with existing literature, participants reported concerns about the confounding effect that hearing loss may have on cognitive assessments which are delivered orally. Previous research has demonstrated that measures of cognition may be underestimated if sensory impairments are not considered or adjusted for (68, 69). Efforts have been made to develop or adapt cognitive tests for people with hearing impairment (70, 71). However, a scoping review of cognitive screening and assessment tools adapted for people with sensory impairment found that the sensitivity and validity of these instruments is poor (44). It is important to keep in mind that screening tools only allow healthcare professionals a snapshot view into an individual's cognitive state at the time of administration, so that the results may be unreliable. Brief cognitive tests, such as the Mini-Mental State Examination (72), often have cut-off scores for determining the presence of cognitive impairment but these should be viewed with caution. Similarly, in this study participants with hearing loss highlighted how it is important to consider patient's hearing status prior to screening, as the results of an oral test may not be accurately represented. There are other factors which may influence test performance including vision impairment, age of participant, level of education and mood. Future research is still required to develop reliable tools for identifying cognitive impairment which take into account the effects of hearing loss.

Previous findings have suggested that identification of cognitive impairment can help inform audiological management in this population, programming of hearing aid devices and settings and longer-term care planning (73); however, this was not reflected in the present study. Most of the support for cognitive screening emerging from this study emphasized that it would potentially enable an earlier diagnosis and thus access to treatment and support which could mitigate further cognitive decline. Results from this study highlight how patients would be keen to ensure longer-term support and follow up if any cognitive impairment were to be discovered which currently may not be readily available. In the UK there is limited post-diagnostic support for people living with MCI/dementia despite evidence-based guidance suggesting the use of interventions to promote cognition, independence and wellbeing (74, 75). Moreover, a recent report by the Alzheimer's Society highlighted how a lack of post-diagnostic support results in more frequent crisis such as health deterioration and hospitalization for the person with dementia as well as carer breakdown (74). More so than ever, post-diagnostic support for people living with dementia has been adversely impacted by the COVID-19 pandemic (76). The lack of post-diagnostic support is similar in National Health Service (NHS) audiology services. In the UK, only half of services offered follow up appointments to their patients, despite two-thirds of patients reporting the need for further support (2).

This study is not without limitations. Participants were recruited purposively, from social media and from a database of individuals who have previously registered their interest in participating in research. Thus, the results may be more representative of adults who are more knowledgeable about hearing research compared to the general population. In addition, the sample was mainly White British (*n* = 87), comprised of mostly retired individuals (*n* = 51) demonstrating a lack of ethnic and sociodemographic diversity. Although this sample is not representative of the age structure of the whole UK population, it does represent an age group that is likely to have most concern about hearing loss and the development of cognitive impairment.

This study also had various strengths. In particular, this was a high-quality qualitative study that was carried out in accordance with best practice recommendations. Specifically, several techniques for enhancing the trustworthiness and rigor of qualitative research were utilized (48, 77). One such technique was qualitative triangulation, or collecting data about the research question using different techniques (i.e., a survey and a workshop). This process also gave us the opportunity to carry out member reflection whereby the workshop participants could reflect and elaborate on their survey responses and provide in-depth feedback on the survey themes. Furthermore, the data analysis was conducted using an established procedure (49, 52). The quality of this analysis was further strengthened via peer debriefing, which included two authors independently analyzing the data and comparing their results and all authors providing feedback on the thematic analysis. We also carried out disconfirming evidence analysis, which entails reporting notable cases that contradict the overall patterns, trends, or themes. For example, we noted that though many participants felt that they would have a negative emotional reaction to cognitive screening, a minority thought that they would be unaffected.

This study demonstrates that although cognitive screening in audiological assessments were generally acceptable to our participants, several changes would be needed before it could be introduced into routine adult audiology practice in future. Indeed, screening for dementia in asymptomatic patients is not currently advised by Public Health England (78), and a change to this recommendation would require a clearer evidence base of benefit. It is also likely, that additional time and staff resources would be necessary to address some of the concerns highlighted in this study. Audiologists may require supplementary training to deliver this form of specialized test for patients with hearing loss. Additional time would be required during appointments to discuss the purpose of and conduct the screening test and explain the results to patients. There are still questions to be raised if cognitive screening were to be embedded in clinical practice. As the scope of this project was to focus on the acceptability of screening for patients, we acknowledge that this is only one consideration in the potential implementation of cognitive screening into audiological clinical practice, and that many other factors would need to be considered to inform potential implementation. Future research should be undertaken to investigate challenges, starting with audiologist perceptions of cognitive screening including their attitudes and beliefs, as well as practical considerations.

## 5. Conclusions

To our knowledge this is the first study to explore patient perceptions on this topic. Although acceptable to patients, the findings suggest that if cognitive screening were to be incorporated into clinical audiology practice, audiologists would require sufficient time within appointments to discuss and explain the rationale for screening as well as information on the potential benefits. Although, evidence to inform best practice is still currently lacking, this study provides a first step toward identifying a patient-centered approach to cognitive screening within audiological care.

## Data availability statement

The raw data supporting the conclusions of this article will be made available by the authors, without undue reservation.

## Ethics statement

This study involving human participants was reviewed and approved by the University of Nottingham Faculty of Medicine and Health Science Research Ethics Committee (FMHS 438-0122). Electronic informed consent to participate in this study was provided by all participants.

## Author contributions

The study was conceived by EB. EB, EH, HH, and TD designed the study. EB and PT collected the data, analyzed the online survey data, and wrote the draft manuscript. EH and PB analyzed and reported the workshop data. All authors critically reviewed and approved the final manuscript.
